# A General Protocol for Synthesizing Thiolated Folate Derivatives

**DOI:** 10.3390/molecules28135228

**Published:** 2023-07-05

**Authors:** Jie Li, Yao Wang, Liangang Shan, Lei Qian, Wenchao Wang, Jixian Liu, Jianguo Tang

**Affiliations:** Institute of Hybrid Materials, National Center of International Research for Hybrid Materials Technology, National Base of International Science & Technology Cooperation, College of Materials Science and Engineering, Qingdao University, Qingdao 266071, China

**Keywords:** folic acid, targeted delivery, sulfhydryl, thiolated folic acid, silver nanoparticles

## Abstract

Folic acid (FA) has shown great potential in the fields of targeted drug delivery and disease diagnosis due to its highly tumor-targeting nature, biocompatibility, and low cost. However, FA is generally introduced in targeted drug delivery systems through macromolecular linkage via complex synthetic processes, resulting in lower yields and high costs. In this work, we report a general protocol for synthesizing thiolated folate derivatives. The small molecule thiolated folate (TFa) was first synthesized with a purity higher than 98.20%. First, S-S-containing diol was synthesized with a purity higher than 99.44 through a newly developed green oxidation protocol, which was carried out in water with no catalyst. Then, folic acid was modified using the diol through esterification, and TFa was finally synthesized by breaking the disulfide bond. Further, the synthesized TFa was utilized to modify silver nanoparticles. The results showed that TFa could be easily bonded to metal particles. The protocol could be extended to the synthesis of a series of thiolated derivatives of folate, such as mercaptohexyl folate, mercaptoundecyl folate, etc., which would greatly benefit the biological applications of FA.

## 1. Introduction

For the past decade, folic acid (FA) [1,2,3] has been attracting considerable attention in the fields of targeted drug delivery [4,5] and cancer therapy [6] because of its tumor-targeting capability and low cost. FA can be integrated with a diverse range of nanomaterials, including metal nanoparticles [7,8,9], polymers [10,11], chitosan [3], liposomes [12], and oxides [13], to fabricate various drug delivery systems. FA is an important biological ligand [14] and a broad-spectrum targeted biological reagent whose receptor (FRα) [15,16] is overexpressed in most tumor tissues [17,18]. Shengnan Huang et al. [19] reported a gene therapy combined with photothermal therapy employing FA-modified gold nanocages for the delivery of anti-MIR-181B and achieved good results in treating hepatocellular carcinoma. Xiaoyu Wang and coworkers [20] fabricated a novel fluorescent probe for detecting cancer cells by combining folic acid-conjugated poly(l-lysine) (FA-PLL) with fluorescent gold nanoclusters. Wu Y and colleagues [21] prepared FA-modified laponite nanodisks for targeted anticancer drug delivery utilizing 3-aminopropy-ldimethylethoxysilane as a linker. To date, FA is generally bound to main matrices through a bridge of bifunctional macromolecules (such as PEG (Thiol)_2_, etc.) or amino acids carrying sulfhydryl groups [22,23]. The aforementioned complex processes resulted in lower purity and high costs. It is worth noting that the length and structure of the linker are key factors in the interaction of folic acid with FR on the cell surface, and it is meaningful to develop new folate derivatives of different molecular chain lengths to determine the types of folate derivatives suitable for biological applications as well as their targeting performance, biocompatibility, and cell toxicity. Sulfhydryl [24] is a bioactive substance that can be bio-coupled with drug carrier molecules such as proteins, DNA, or metal nanoprobes [25], so conceiving the small molecule thiolated folic acid (TFa) with different molecular chain lengths and structures would greatly facilitate applications of FA [26]. It is the key to introducing the sulfhydryl group into the FA molecule chain because of its high chemical activity, which is higher than that of the hydroxyl and amine groups. It is a good scheme to modify folic acid with disulfide (SS) and then break the disulfide bond to form TFa [27,28]. To date, disulfide is generally synthesized by oxidizing sulfides, which is carried out in organic solvents employing transition metal catalysts and hydrogen peroxide, chlorine, or oxygen as oxidants. M.M. Khodaei et al. [29] oxidized 2-Hydroxy-1-ethanethiol to SS using acetonitrile as a solvent, Bi (NO_3_)_3_ as a catalyst, and air as an oxidant. Makoto Oba et al. [30] reported the oxidation of 2-Hydroxy-1-ethanethiol to SS, adopting dichloromethane as a solvent and bis(4-methoxyphenyl) telluride as a catalyst. The above oxidants are stronger to lead to byproducts such as isethionate, and the residue of the solvent and the heavy metal catalysts is also not conducive to subsequent biosynthesis. Moreover, the purity of commercial SS is only about 90%, which is not suitable for the synthesis of TFa because produced folic acid derivatives are very difficult to separate and purify. Zeynizadeh Behzad reported his work on the oxidative coupling of thiols to disulfides, employing iodine as an oxidant and wet acetonitrile as a solvent, and obtained a higher yield of 94% [31]. Here, we developed a green routine using iodine as the oxidant and pure water as the solvent to prepare S-S-containing diol without catalysts, which was helpful for the purification of subsequent products. The purity of the obtained S-S-containing diol was 99.44%. Then, SS-modified FA (Fa-SS) was prepared through esterification, and thiolated folate (TFa) was finally synthesized by breaking the disulfide bond of Fa-SS using dithiothreitol (DTT) as a reductant. The purity of the synthesized TFa was higher than 98.20%. Further, the resultant TFa was used to modify silver nanoparticles, and the results showed that TFa could be easily bonded to noble metal particle surfaces such as Ag, Au, etc.

## 2. Results and Discussion

### 2.1. Synthesis of Dithiobisethanol (SS)

**Figure 1:**
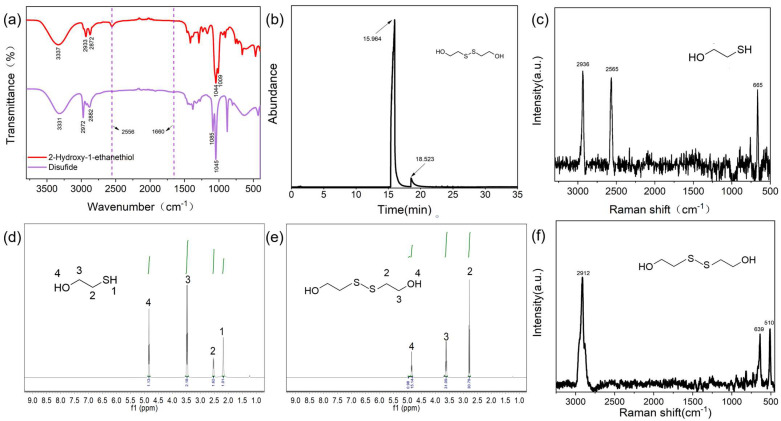
Characterization of 2-Hydroxy-1-ethanethiol and the synthesized SS in the work: (**a**) near-infrared spectra of 2-Hydroxy-1-ethanethiol and SS; (**b**) gas chromatography spectrum of SS; (**c**) Raman spectrum of 2-Hydroxy-1-ethanethiol; (**d**) nuclear magnetic resonance spectrum of 2-Hydroxy-1-ethanethiol; (**e**) nuclear magnetic resonance spectrum of SS; (**f**) Raman spectrum of SS.

In order to synthesize high-purity disulfide, controlling the conversion rate and the selectivity of the reaction is the key. In this work, iodine, a mild oxidant, was employed as an oxidant and water was employed as a solvent to reduce the excessive oxidation of sulfhydryl and the contamination of products by organic solvents. The as-synthesized SS was characterized using the near-infrared spectrum (Section S2.2 in the Supplementary Materials), the gas chromatography spectrum (Section S2.6 in the Supplementary Materials), and nuclear magnetic resonance spectroscopy (Section S2.4 in the Supplementary Materials) (Figure 1). In the IR spectrum of SS (Figure 1a), the characteristic absorption peak of the H-S bond at 2556 cm^−1^ disappeared compared to that of 2-Hydroxy-1-ethanethiol, indicating that the sulfhydryl group was transformed to a S-S bond. The conversion rate of the reaction was 83.16%, based on the UV-Vis spectrum (Section S2.1 in the Supplementary Materials) (Figure S1), and the yield of SS was 70.13% (0.108 g/0.154 g × 100% = 70.13%). In the gas chromatography–mass spectrometry (GC-MS) analysis (Section S2.6 in the Supplementary Materials), the main byproduct was 4.65% 2,2′-trithiobisethanol, and the reaction selectivity was 94.79% for the production of SS (Figure 1b and Table S1). Because 2,2′-trithiobisethanol has a similar molecular structure and reactivity to SS and no effect on the subsequent reactions and final products, 2,2′-trithiobisethanol can also be counted as a product of SS. Therefore, the total apparent selectivity of the reaction can be estimated as 99.44%. We performed a H NMR analysis (SI Section S2.4). In the H NMR spectra (Figure 1e) of SS, the characteristic proton peak of the S-H bond at 2.18 ppm disappeared compared to that of 2-Hydroxy-1-ethanethiol (Figure 1d), indicating that sulfhydryl did not exist in the disulfides. The transformation of the sulfhydryl group to disulfide was further verified with Raman spectroscopy (Section S2.3 in the Supplementary Materials). It can be seen from Figure 1c, f that the characteristic peak of the S-H bond at 2565 cm^−1^ appeared in the Raman spectrum of 2-Hydroxy-1-ethanethioll but disappeared in the Raman spectrum of SS, and a new peak at 510 cm^−1^ appeared in the latter, which was the characteristic peak of the S-S bond. Compared with 2-Hydroxy-1-ethanethiol, in the latter, the characteristic CH peak shifted slightly from 2936 cm^−1^ to 2912 cm^−1^, and the characteristic C-S peak shifted slightly from 665 cm^−1^ to 639 cm^−1^. The result further identified that the sulfhydryl group of 2-Hydroxy-1-ethanethiol was successfully transformed into a S-S bond.

Based on our experimental findings, increasing the iodine content by 0.5 times can produce a conversion rate of 100% for 2-Hydroxy-1-ethanethiol but leads to more byproducts such as isethionic acid. To avoid potential negative effects on the subsequent reaction with folic acid, we determined the ratio of iodine to 2-Hydroxy-1-ethanethiol is 1:2. The purity of the isolated SS was higher than 99.44% based on GC-MS (Figure 1b), which was higher than that reported in the pioneering literature (Table S2). In previous works [27,28,29,30,31] using O_2_, Cl_2_, H_2_O_2,_ or I_2_ as oxidants, there were always byproducts formed during the reactions, such as isethionate, resulting in lower purity, and the residual catalysts and organic solvents caused difficult separation and affected the subsequent esterification (Table S2). It should be noted that the protocol is an environmentally friendly process without an organic solvent or catalyst, and the reaction solution can be recovered for a further reaction after product separation, which assures the reaction has a higher yield and purity.

### 2.2. Synthesis of S-S-Containing Diol-Modified FA (Fa-SS)

**Figure 2:**
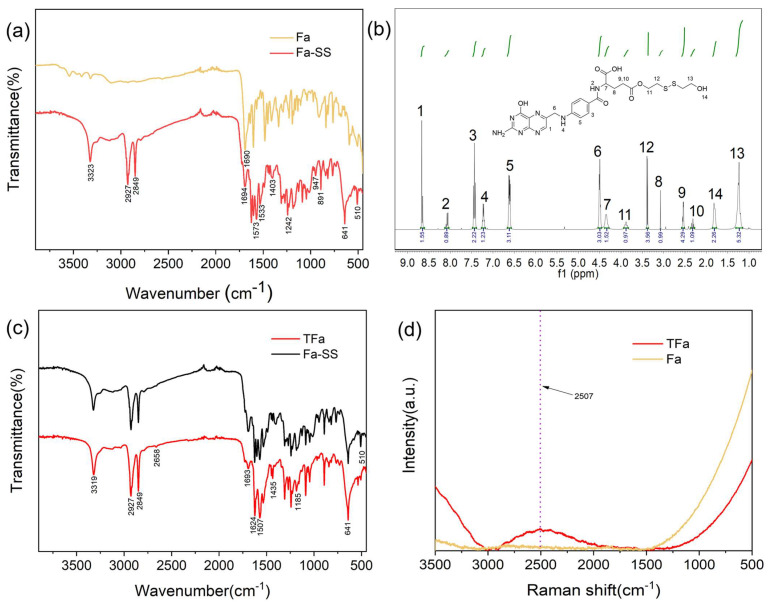
Characterization of FA and the synthesized Fa-SS and TFa in this work. (**a**) Near-infrared spectra of FA and Fa-SS. (**b**) Nuclear magnetic resonance spectrum of Fa-SS. (**c**) Near-infrared spectra of Fa-SS and TFa; (**d**) Raman spectra of FA and TFa.

S-S-containing diol-modified FA (Fa-SS) was synthesized through the esterification of FA with S-S-containing diol. FA and the synthesized Fa-SS were tested with IR spectroscopy (Section S2.2 in the Supplementary Materials) (Figure 2a). In the IR spectrum of Fa-SS, there were three obviously enhanced peaks at 3338 cm^−1^, 2927 cm^−1^, and 2849 cm^−1^ compared to that of FA, which belonged to the stretching vibration absorption peaks of the O-H and C-H bonds of SS, respectively. The results implied that SS molecules were successfully connected with FA and one O-H group, and four methylene groups were introduced in each Fa-SS molecular chain, increasing their absorption peak. The peak at 1694 cm^−1^ was still the stretching vibration absorption peak of C=O; the peaks at 1573 cm^−1^ and 1533 cm^−1^ were the skeletal vibration absorption peaks of the benzene ring; and the peaks at 1403 cm^−1^ and 1242 cm^−1^ were the deformation vibration absorption peaks of CH_2_. The peak at 947 cm^−1^ was the stretching vibration absorption peak of C-H on the heterocycles, and the peak at 891 cm^−1^ was the stretching vibration absorption peak of C-H on the benzene ring. Moreover, two new sets of peaks appeared: the absorption peak at 641 cm^−1^ was assigned to the stretching vibration of C-S and the absorption peak at 510 cm^−1^ was assigned to the stretching vibration of S-S, both of which originated from SS, further indicating the successful conjugation of SS with FA.

We performed a H NMR analysis (Section S2.4 in the Supplementary Materials). In the NMR spectrum of Fa-SS (Figure 2b), there were four new proton absorption peaks, namely, δ 3.88 ppm, 3.38 ppm, 1.82 ppm, and 1.23 ppm, which belonged to the H of the -CH_2_- linked to the ester bond, the H of the -CH_2_- linked to -S-S, the H of the -CH_2_- linked to -OH, and the H of the -OH from CH_2_-CH_2_-OH, respectively. The three very small peaks at 2.42 ppm, 2.95 ppm, and 5.34 ppm could have been derived from the residual DCU in the products and the impurities of the residual solvent (DMSO) used in the process. The results further verified that the ester bond between FA and SS formed. Fa-SS was successfully prepared, and the yield was 79.07% (0.457 g/0.578 g × 100% = 79.07%).

### 2.3. Synthesis of TFa

TFa was prepared by breaking the disulfide bond of Fa-SS, employing dithiothreitol (DTT) as a reductant. The infrared spectrum of the synthesized TFa (Section S2.2 in the Supplementary Materials) is shown in Figure 2c, in which the S-H stretching vibration absorption peak at 2658 cm^−1^ appeared, while the S-S absorption peak at 510 cm^−1^ disappeared, indicating that the S-S bond was transformed to a S-H bond. The peak at 1693 cm^−1^ was the absorption peak of carbonyl C=O; the peaks at 1624 cm^−1^ and 1507 cm^−1^ belonged to the skeleton vibration absorption bands of the benzene ring; the peak at 1435 cm^−1^ was attributed to the absorption peak of methylene CH_2_; and the strong peak at 1185 cm^−1^ was the absorption peak of the C-O-C of the formed ester bond. The vibration absorption peak of the C-S bond was at 641 cm^−1^. In the Raman spectra of the synthesized TFa (Section S2.3 in the Supplementary Materials) (Figure 2d), a prominent characteristic peak appeared at 2507 cm^−1^ compared to that of FA, which was the characteristic absorption peak of the S-H stretching vibration. We performed a H NMR analysis (SI Section S2.4). The H NMR results further indicated that TFa was successfully prepared by breaking the disulfide bond of Fa-SS (Figure S2). The purity of the synthesized TFa was higher than 98.20%, which could be further identified with HLPC (Section S2.5 in the Supplementary Materials) (Figure S3). The results also showed that the conversion rate of TFa was 73.19% (Section S2.1 in the Supplementary Materials) (Figure S4), and the yield was 65.74% (0.165 g/0.251 g × 100% = 65.74%), much higher than the reported data [22,23], in which the effective conjugation rate of FA was lower than 30%.

### 2.4. Modification of Silver Nanoparticles (AgNPs) Using TFa

**Figure 3:**
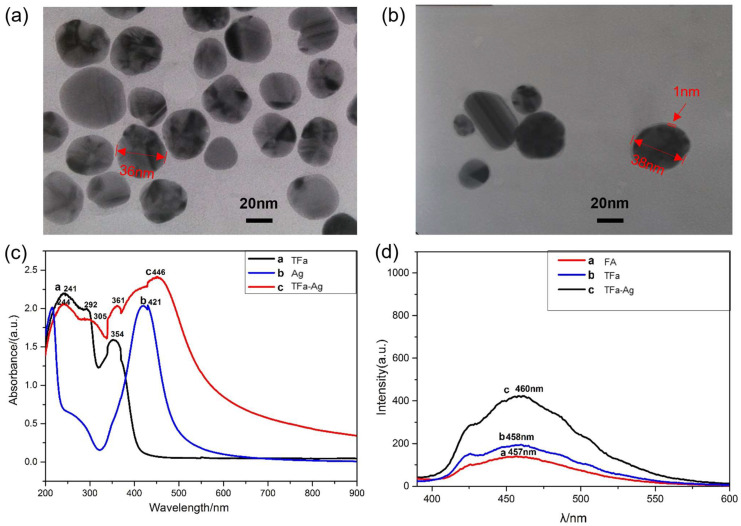
(**a**) TEM image of AgNPs. (**b**) TEM image of TFa-modified AgNPs. (**c**) UV-Vis spectra of samples. (**d**) Fluorescent emission spectra of samples.

Modifying silver nanoparticles (AgNPs) was performed by mixing the ethanol solution of TFa and a AgNP–water dispersion, during which the surfaces of the AgNPs were capped by TFa molecules via molecular self-assembly. Figure 3a,b show transmission electron microscopy (TEM) images of AgNPs before and after modification (Section S2.7 in the Supplementary Materials). The images show that a thin film of about 1 nm thickness formed on the surfaces of the AgNPs after the modification, which was further identified as a TFa molecule layer with FTIR spectroscopy (Figure S5 in the Supplementary Materials). The results indicated that TFa could be easily bonded to metal particles and used as a targeting reagent in bio-pharmaceuticals and bio-probes.

AgNPs were modified with thiolated folic acid, and the products were separately dissolved in a 1.7 mM dilute ammonia aqueous solution with pH = 8 to obtain 3 mmol/L samples and were subjected to UV (Figure 3c and Section S2.6 in the Supplementary Materials). The spectra showed that TFa had three sets of UV absorption peaks, i.e., 241 nm, 292 nm, and 354 nm; AgNPs had one UV-Vis absorption peak at 421 nm; and TFa-modified AgNPs contained the main absorption peaks of TFa and AgNPs, indicating that TFa molecules were successfully modified on the surfaces of the silver nanoparticles. At the same time, because the particle size of the modified AgNPs increased, the absorption peak of AgNPs red-shifted from 421 nm to 446 nm, and the absorption peaks of capped TFa also red-shifted to 244 nm, 292 nm, and 361 nm, respectively.

Folic acid has weak fluorescence, and the luminescence properties of modified silver nanoparticles have been studied. A fluorescence spectrophotometer separately tested FA, TFa, and TFa-modified AgNPs. The products were dissolved separately in a dilute ammonia solution with pH = 8, prepared to 10 mmol/L. An excitation wavelength of 370 nm was selected, and fluorescence testing was performed at 25 °C (Section S2.8 in the Supplementary Materials), as shown in Figure 3d. When the excitation wavelength was 370 nm, the fluorescence emission peak of FA appeared at 457 nm, and the fluorescence emission peak of TFa was slightly red-shifted to 458 nm. TFa-modified AgNPs had a strong fluorescence emission peak at 460 nm, which was significantly higher than that of FA and TFa, indicating that TFa-modified AgNPs have potential applications in the field of bio-diagnostics and biological probes.

## 3. Materials and Methods

### 3.1. Materials

Folic acid, 2-Hydroxy-1-ethanethiol, and dimethyl sulfoxide (DMSO) were purchased from Tianjin Guangcheng Chemical Reagent Co., Ltd. (Tianjin, China); ethanol, ethyl acetate, sodium hydroxide, oleylamine (OA), isooctane, ascorbic acid, bis (2-ethylhexyl) sulfosuccinate sodium (AOT, 98.0%), and dithiothreitol (DTT) were obtained from Macklin reagents Co., Ltd. (Shanghai, China); and sodium borohydride, Polyvinylpyrrolidone (PVP), silver nitrate, 4-dimethylaminopyridine (DMAP), Dicyclohexylcarbodiimide (DCC), and ammonia water were purchased from Sinopharm Chemical Reagent Co., Ltd. (Tianjin, China). All reagents were analytically pure and were used as received.

### 3.2. Synthesis of Disulfide: 2,2′-Dithiobis-Ethanol (SS)

**Figure 4:**
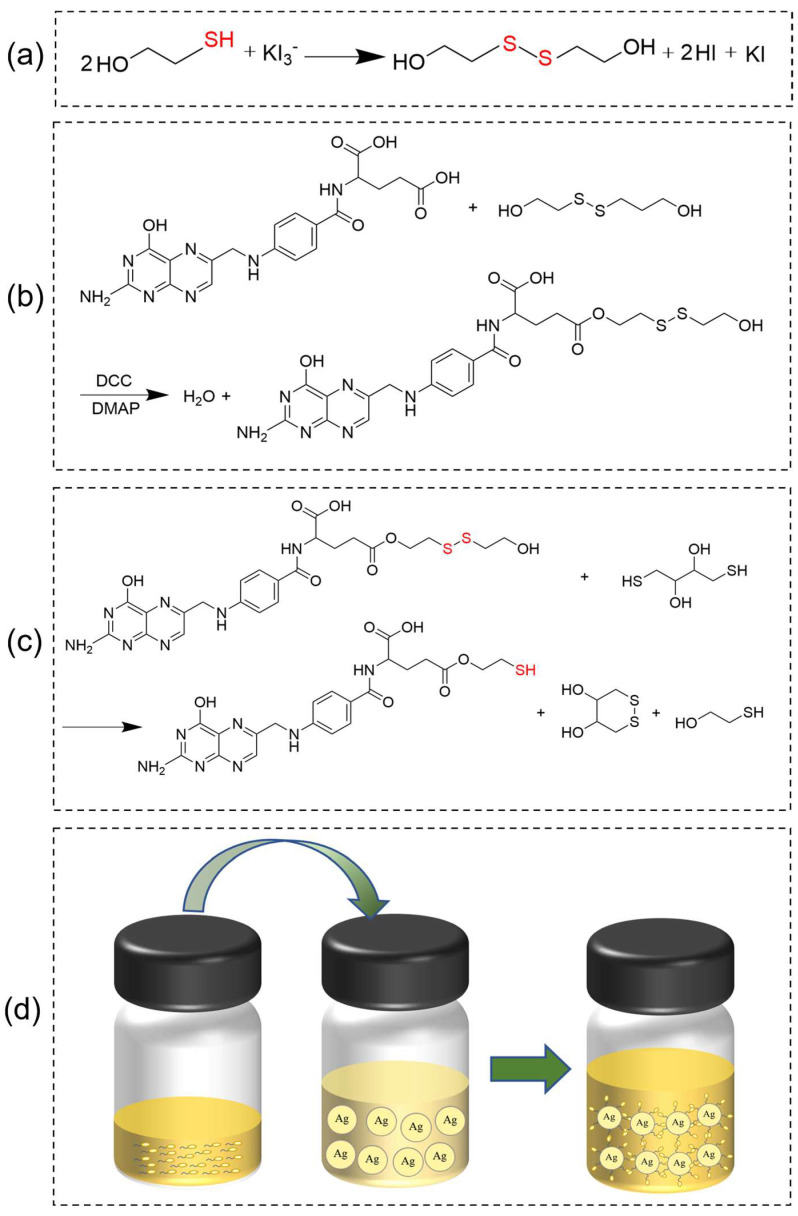
Synthetic scheme. (**a**) Synthesis of disulfide (SS) from 2-Hydroxy-1-ethanethiol. (**b**) Synthesis of S-S-containing diol-modified FA (Fa-SS) via esterification. (**c**) Preparation of thiolated folic acid (TFa). (**d**) Modification of silver nanoparticles (AgNPs) using TFa.

Disulfide (SS) was synthesized via the oxidation of 2-Hydroxy-1-ethanethiol, employing iodine as an oxidant. The reaction formula is shown in Figure 4a. First, 1 mmol iodine was added to 10 mL of an aqueous solution of 4 mM potassium iodide and stirred until the iodine was thoroughly dissolved. Then, 2 mmol 2-Hydroxy-1-ethanethiol was added to the above solution, and the reaction continued for two hours. Subsequently, 10 mL of ethyl acetate was added to the yellow reaction mixture and stirred for 15 min. The upper layer, the ethyl acetate solution of SS, was obtained via static layering. The disulfide was obtained via rotary evaporation. The melting point of SS is 25 °C (Section S2.9 in the Supplementary Materials), and the yield of SS was 70.13%.

### 3.3. Synthesis of S-S-Containing Diol-Modified FA: 2,2′-Dithiobis-Ethyl Folate (Fa-SS)

Fa-SS was prepared via the esterification of FA with S-S-containing diol. The reaction formula is shown in Figure 4b. Typically, 1 mmol FA (0.441 g), 1 mmol DCC (0.206 g), and 0.3 mmol DMAP (0.036 g) were separately added to a 100 mL round-bottom flask containing 20 mL of DMSO in a 37 °C water bath and stirred until the solution became clear. Then, 1 mmol S-S-containing diol (0.154 g) was added to the solution, and the reaction was carried out for 12 h at 37 °C under stirring, during which the solution gradually became turbid, and the mixture was filtered to remove the N,N′-Dicyclohexylurea (DCU). Next, 40.0 mL of ethyl acetate and 160.0 mL of distilled water were added to the filtrate containing Fa-SS under stirring, during which Fa-SS was precipitated in the form of an orange-yellow flocculent, collected via filtration, and washed twice with 5 mL of ethanol and 5 mL of diethyl ether, respectively. Finally, Fa-SS was obtained via freeze drying. The melting point of Fa-SS is 293.9 °C (Figure S6a in the Supplementary Materials), and the yield of Fa-SS was 79.07%.

### 3.4. Synthesis of Thiolated Folate Derivatives: Thiol-Ethyl Folate (TFa)

TFa was prepared by reducing Fa-SS, employing dithiothreitol (DTT) as a reductant. The reaction formula is shown in Figure 4c. First, 0.5 mmol Fa-SS (0.289 g) was dissolved in 15 mL of DMSO. Then, 0.5 mmol DTT (0.077 g) was added to the solution, and the reaction was carried out for 10 h under stirring at 37 °C. After the reaction, 30.0 mL of ethyl acetate and 150.0 mL of distilled water were added to the reaction solution under stirring, during which TFa was precipitated in the form of a yellow flocculent, collected via filtration, and washed twice with 5 mL of ethanol and 5.0 mL of diethyl ether, respectively. Finally, TFa was obtained via freeze drying. The melting point of Fa-SS is 296.3 °C (Figure S6b in the Supplementary Materials), and the yield of TFa was 65.74%.

### 3.5. Modification of Silver Nanoparticles (AgNPs) Using TFa

AgNPs were synthesized using the method we reported previously [32]. The reaction flowchart is shown in Figure 4d. Typically, 0.5 mmol bis (2-ethylhexyl) sulfosuccinate sodium (AOT, 0.223 g) was first dissolved in 5 mL of isooctane and stirred. Then, 90.0 µL of a 0.3 M aqueous ascorbic acid solution was added to the above AOT solution, and the mixture was ultrasonically emulsified until a transparent microemulsion formed. Next, 250 µL of an isooctane solution containing 10.0 mM Ag(I) was added dropwise to the microemulsion, and the mixture turned yellowish. The reaction was carried out for 1.0 h, and silver nanoparticles were obtained with a size of approximately 36.0 nm (Figure 3a). After that, 10 µL of a 0.1 M TFa–dilute ammonia aqueous solution (the concentration of dilute ammonia water was 1.7 mM, and the pH value was about 8.0) was added to the mixture and stirred for 100 min, which allowed the TFa to be conjugated on the surfaces of the silver nanoparticles. The products were separated by adding an equal amount of water. The mixture turned nontransparent, and a white emulsion formed immediately. Then, the emulsion was left in the refrigerator at 4 °C for 10 min, and a black powder could be seen depositing on the bottom of the bottle. The powder was collected through centrifugation at 3000 rpm for 5.0 min, and the collected products were dispersed in 5 mL of ethanol, forming a yellow transparent solution.

## 4. Conclusions

In this work, TFa was first prepared using a simple three-step process, and the structures of the products were characterized and verified with IR, NMR, etc. The results indicated that the final product was successfully prepared with a purity higher than 98.20%. The method is easy to handle with a high yield and purity, and the synthesized TFa can be easily bound to metal particles, implying that the final product can be directly used as a targeting reagent in drug delivery systems, disease diagnosis, and cancer therapy, especially in those containing metal nanoparticles. New folate derivatives of different molecular chain lengths will be meaningful in determining the impact of the length and structure of the linker upon the interaction of folic acid with FR. The method also provides great potential for fabricating the derivatives of other organics and provides a new technique for the biological application of folic acid.

## Data Availability

The data are contained within the article or supplementary material.

## Supplementary Materials

The following supporting information can be downloaded at: https://www.mdpi.com/article/10.3390/molecules28135228/s1, Figure S1: Conversion and yield of SS; Figure S2: H NMR spectra of FA, Fa-SS, and TFa; Figure S3: Yield of TFa; Figure S4: UV-Vis of FA, Fa-SS, and TFa; Figure S5: FTIR spectrum of TFa-modified AgNPs; Figure S6: Melting point of Fa-SS and TFa; Table S1: GC-MS data of the synthesized SS; Table S2: Yield data from the literature; Table S3: The data from the HPLC pattern of FA; Table S4: The data from the HPLC pattern of TFa.

## References

[1] Ebrahimnejad P., Sodagar Taleghani A., Asare-Addo K., Nokhodchi A. (2022). An updated review of folate-functionalized nanocarriers: A promising ligand in cancer. Drug Discov. Today.

[2] Buskaran K., Bullo S., Hussein M.Z., Masarudin M.J., Mohd Moklas M.A., Fakurazi S. (2021). Anticancer Molecular Mechanism of Protocatechuic Acid Loaded on Folate Coated Functionalized Graphene Oxide Nanocomposite Delivery System in Human Hepatocellular Carcinoma. Materials.

[3] Zhong S., Zhang H., Liu Y., Wang G., Shi C., Li Z., Feng Y., Cui X. (2017). Folic acid functionalized reduction-responsive magnetic chitosan nanocapsules for targeted delivery and triggered release of drugs. Carbohydr. Polym..

[4] Khan M.S., Talib A., Pandey S., Bhaisare M.L., Gedda G., Wu H.F. (2017). Folic Acid navigated Silver Selenide nanoparticles for photo-thermal ablation of cancer cells. Colloids Surf. B Biointerfaces.

[5] Liu Y., Zong Y., Yang Z., Luo M., Li G., Yingsa W., Cao Y., Xiao M., Kong T., He J. (2019). Dual-Targeted Controlled Delivery Based on Folic Acid Modified Pectin-Based Nanoparticles for Combination Therapy of Liver Cancer. ACS Sustain. Chem. Eng..

[6] Fekry A.M., Abdel-Gawad S.A., Tammam R.H., Zayed M.A. (2020). An electrochemical sensor for creatinine based on carbon nanotubes/folic acid/silver nanoparticles modified electrode. Measurement.

[7] Jesna K.K., Ilanchelian M. (2017). Photophysical changes of thionine dye with folic acid capped gold nanoparticles by spectroscopic approach and its in vitro cytotoxicity towards A-549 lung cancer cells. J. Mol. Liq..

[8] Khademi S., Sarkar S., Shakeri-Zadeh A., Attaran N., Kharrazi S., Ay M.R., Ghadiri H. (2018). Folic acid-cysteamine modified gold nanoparticle as a nanoprobe for targeted computed tomography imaging of cancer cells. Mater. Sci. Eng. C Mater. Biol. Appl..

[9] Papaioannou L., Angelopoulou A., Hatziantoniou S., Papadimitriou M., Apostolou P., Papasotiriou I., Avgoustakis K. (2018). Folic Acid-Functionalized Gold Nanorods for Controlled Paclitaxel Delivery: In Vitro Evaluation and Cell Studies. AAPS PharmSciTech.

[10] Wang J., Xu T. (2011). Facile construction of multivalent targeted drug delivery system from Boltorn^®^ series hyperbranched aliphatic polyester and folic acid. Polym. Adv. Technol..

[11] Cheng W., Nie J., Xu L., Liang C., Peng Y., Liu G., Wang T., Mei L., Huang L., Zeng X. (2017). pH-Sensitive Delivery Vehicle Based on Folic Acid-Conjugated Polydopamine-Modified Mesoporous Silica Nanoparticles for Targeted Cancer Therapy. ACS Appl. Mater. Interfaces.

[12] Chiani M., Norouzian D., Shokrgozar M.A., Azadmanesh K., Najmafshar A., Mehrabi M.R., Akbarzadeh A. (2018). Folic acid conjugated nanoliposomes as promising carriers for targeted delivery of bleomycin. Artif. Cells Nanomed. Biotechnol..

[13] Huang Y., Mao K., Zhang B., Zhao Y. (2017). Superparamagnetic iron oxide nanoparticles conjugated with folic acid for dual target-specific drug delivery and MRI in cancer theranostics. Mater. Sci. Eng. C Mater. Biol. Appl..

[14] Li Y.-A., Zhao X.-D., Yin H.-P., Chen G.-J., Yang S., Dong Y.-B. (2016). A drug-loaded nanoscale metal–organic framework with a tumor targeting agent for highly effective hepatoma therapy. Chem. Commun..

[15] Chen C., Ke J., Zhou X.E., Yi W., Brunzelle J.S., Li J., Yong E.-L., Xu H.E., Melcher K. (2013). Structural basis for molecular recognition of folic acid by folate receptors. Nature.

[16] Soleymani J., Hasanzadeh M., Somi M.H., Shadjou N., Jouyban A. (2018). Probing the specific binding of folic acid to folate receptor using amino-functionalized mesoporous silica nanoparticles for differentiation of MCF 7 tumoral cells from MCF 10A. Biosens. Bioelectron..

[17] Nosrati H., Abbasi R., Charmi J., Rakhshbahar A., Aliakbarzadeh F., Danafar H., Davaran S. (2018). Folic acid conjugated bovine serum albumin: An efficient smart and tumor targeted biomacromolecule for inhibition folate receptor positive cancer cells. Int. J. Biol. Macromol..

[18] Wang H., Lin S., Wang S., Jiang Z., Ding T., Wei X., Lu Y., Yang F., Zhan C. (2022). Folic Acid Enables Targeting Delivery of Lipodiscs by Circumventing IgM-Mediated Opsonization. Nano Lett..

[19] Huang S., Duan S., Wang J., Bao S., Qiu X., Li C., Liu Y., Yan L., Zhang Z., Hu Y. (2016). Folic-Acid-Mediated Functionalized Gold Nanocages for Targeted Delivery of Anti-miR-181b in Combination of Gene Therapy and Photothermal Therapy against Hepatocellular Carcinoma. Adv. Funct. Mater..

[20] Wang X., Xia J., Wang C., Liu L., Zhu S., Feng W., Li L. (2017). Preparation of Novel Fluorescent Nanocomposites Based on Au Nanoclusters and Their Application in Targeted Detection of Cancer Cells. ACS Appl. Mater. Interfaces.

[21] Wu Y., Guo R., Wen S., Shen M., Zhu M., Wang J., Shi X. (2014). Folic acid-modified laponite nanodisks for targeted anticancer drug delivery. J. Mater. Chem. B.

[22] Kumar S.S.D., Mahesh A., Antoniraj M.G., Rathore H.S., Houreld N.N., Kandasamy R. (2018). Cellular imaging and folate receptor targeting delivery of gum kondagogu capped gold nanoparticles in cancer cells. Int. J. Biol. Macromol..

[23] Liu Z., Turyanska L., Zamberlan F., Pacifico S., Bradshaw T.D., Moro F., Fay M.W., Williams H.E.L., Thomas N.R. (2019). Synthesis of folic acid functionalized gold nanoclusters for targeting folate receptor-positive cells. Nanotechnology.

[24] Delarue Bizzini L., Zwick P., Mayor M. (2019). Preparation of Unsymmetrical Disulfides from Thioacetates and Thiosulfonates. Eur. J. Org. Chem..

[25] Abbasian M., Judi M., Mahmoodzadeh F., Jaymand M. (2018). Synthesis and characterization of a pH- and glucose-responsive triblock copolymer via RAFT technique and its conjugation with gold nanoparticles for biomedical applications. Polym. Adv. Technol..

[26] Yu Y., Liu J., Tang J., Wang Y., Yue L., Qiu W., Sun Y., Huang Z. (2012). Preparation and characterization of thiolated folic acid. Guangzhou Chem..

[27] Yi M.C., Khosla C. (2016). Thiol–Disulfide Exchange Reactions in the Mammalian Extracellular Environment. Annu. Rev. Chem. Biomol. Eng..

[28] Kim J., Movassaghi M. (2009). Biogenetically inspired syntheses of alkaloid natural products. Chem. Soc. Rev..

[29] Mohammadpoor-Baltork I., Khodaei M.M., Nikoofar K. (2003). Bismuth(III) nitrate pentahydrate: A convenient and selective reagent for conversion of thiocarbonyls to their carbonyl compounds. Tetrahedron Lett..

[30] Oba M., Tanaka K., Nishiyama K., Ando W. (2011). Aerobic Oxidation of Thiols to Disulfides Catalyzed by Diaryl Tellurides under Photosensitized Conditions. J. Org. Chem..

[31] Zeynizadeh B. (2002). Oxidative Coupling of Thiols to Disulfides with Iodine in Wet Acetonitrile. J. Chem. Res..

[32] Liu Z., Xue J., Wang Y., Liu F., Zhou X., Liu J., Tang J. (2020). Silver-Alkylamine Complex Mediated Single Micelle toward Synthesis of Sub-8 nm Silver Nanocrystals. Part. Part. Syst. Charact..

