# Supplementary material for: A General Protocol for Synthesizing Thiolated Folate Derivatives
Source: Molecules. 2023 Jul 5;28(13):5228. doi: 10.3390/molecules28135228 (PMC10343342; doi:10.3390/molecules28135228)
Supplement: Supplementary file 1 [file molecules-28-05228-s001.zip › molecules-2473881-supplementary.pdf]

Supplementary Materials for

A General Protocol for Synthesizing Thiolated Folate Derivatives

Jie Li †, Yao Wang †, Liangang Shan, Lei Qian, Wenchao Wang, Jixian Liu *, Jianguo Tang *

Institute of Hybrid Materials, National Center of International Research for Hybrid Materials Technology, National Base of International Science & Technology Cooperation, College of Materials Science and Engineering, Qingdao University, Qingdao, 266071, China

* Correspondence: ljx@qdu.edu.cn (J.L.); jianguotangde@hotmail.com (J.T.)

† These authors contributed equally to this work.

Table of Content

1 Materials 1
2 Characterization 1
2.1 Ultraviolet-Visible Spectroscopy (UV-Vis) 1
2.2 Fourier Transform Infrared Spectroscopy (FT-IR) 1
2.3 Raman Spectroscopy 2
2.4 NMR Spectroscopy 2
2.5 U3000 HPLC 2
2.6 Gas chromatography-mass spectrometry (GC-MS) 2
2.7 JEM-1200EX TEM 3
2.8 Fluorescence spectroscopy (PL) 3
2.9 Differential scanning calorimetry (DSC) 3
3 Supplementary data 4
3.1 GC-MS 4
3.2 Conversion of SS 5
3.3 Purity data from literatures 6
3.4 H NMR 6
3.5 Purity of TFa 7
3.6 Conversion of TFa 8
3.7 FTIR spectrum of TFa modified AgNPs 9
3.8 Melting point of Fa-SS and TFa 10

1 Materials

Folic acid, 2-Hydroxy-1-ethanethiol and dimethyl sulfoxide (DMSO) were purchased from Tianjin Guangcheng Chemical Reagent Co., Ltd.; ethanol, ethyl acetate, sodium hydroxide ether, oleylamine (OA), isooctane, ascorbic acid, docusate sodium (AOT, 98.0%) and dithiothreitol (DTT) were obtained from Macklin reagents Co., Ltd.; sodium borohydride, Polyvinylpyrrolidone (PVP), silver nitrate, 4-dimethylaminopyridine (DMAP), Dicyclohexylcarbodiimide (DCC) and ammonia water were purchased from Sinopharm Chemical Reagent Co., Ltd. All reagents were analytically pure and used as received.

2 Characterization

2.1 Ultraviolet-Visible Spectroscopy (UV-Vis)

Ultraviolet-Visible Spectrometer (UV-Vis) analysis was conducted using a PerkinElmer-Lambda 750 S instrument. Before the test the instrument was preheated for 20 minutes, and the scanning wavelength range was set to 200-800 nm. The samples and reference signals are separately corrected by their respective black zone signals. Distilled water and ethanol are used as reference samples respectively, and the samples are diluted to an appropriate concentration to avoid exceeding the detection limit of the instrument.

2.2 Fourier Transform Infrared Spectroscopy (FT-IR)

Fourier Transform Infrared Spectroscopy (FT-IR) was conducted using Fourier transform infrared spectrometer (Nicolet IS50 FT-IR, USA). A baseline is first established by taking air as the test background before the test, and the test of liquid samples is carried out via the mode of total reflection.

2.3 Raman Spectroscopy

Raman Spectroscopy was performed using a DXR2 micro-Raman spectrometer (Thermo Fisher Scientific Co., Ltd., USA). The instrument was calibrated before analysis, with laser energy set to 1.5, aperture at 50 μm slit, and an objective lens at 10x magnification. Acquisition time was set to 2, with 32 scans performed. Samples were placed on a coverslip and flattened for analysis using a 532 nm laser kit.

2.4 NMR Spectroscopy

Superconducting nuclear magnetic resonance spectroscopy (NMR) was conducted using a Bruker AVANCE III HD 400 MHz superconducting NMR spectrometer (Germany) with a host power of 7 kW and a maximum speed of 280 rpm, equipped with an encoded torque sensor (patented product). The samples are dissolved in deuterated DMSO and placed in NMR tubes before analysis.

2.5 U3000 HPLC

Another HPLC analysis was performed using a Thermo Fisher U3000 HPLC instrument with a C18 column (250mm×4.6mm, 5 μm). The mobile phase consisted of A: 10 mM ammonium acetate aqueous solution and B: 10 mM ammonium acetate 95:5 acetonitrile/water solution. Chromatographic conditions were set as follows: column temperature at 30°C, injection volume at 10 μL, the flow rate at 0.5 mL/min, and UV detector set at a wavelength of 280 nm.

2.6 Gas chromatography-mass spectrometry (GC-MS)

Gas chromatography-mass spectrometry (GC-MS) uses an Agilent 7890A GC-5975C MS instrument. Mobile phase: argon; flow rate: 30.00 mL/min; column temperature: 190°C.

2.7 JEM-1200EX TEM

The morphology was analyzed by JEM-1200EX electron microscope. Dissolve the product in ethanol solution, ultrasonically disperse it evenly, then take a sample and drop it on a copper grid to dry it for testing.

2.8 Fluorescence spectroscopy (PL)

The fluorescence spectra of the samples gotten from our experiments were tested using Fluorescence spectroscopy (ARL-X1500, Thermo Fisher Scientific Co., Ltd., USA). The slit was set to 10nm and excitation wavelength is 370nm.

2.9 Differential scanning calorimetry (DSC)

Differential scanning calorimetry was used to test the melting point of the product by TA Q20 from USA, and a sample of 10 mg was weighed and placed in an aluminum crucible and put into a test cell for testing. Temperature control program setting: heating from 50 °C to 150 °C at 10 °C/min; holding for 1 min; cooling down to 50 °C at 30 °C/min; holding for 1 min; heating to 320 °C at 10 °C/min.

3 Supplementary data

3.1 GC-MS

Table S1. GC-MS data of the synthesized SS

Retention Time (min)	Area (Ab*s)	Match name	Qualitative	Molecular weight (amu)	Quantitative/%
1.06	2669603	Trisilane, 1,1,1,3,3,3-hexafluoro-	9	199.94	0.03
1.25	1637334	n-Hexane	78	86.11	0.02
1.33	3019386	Thiirane	53	60	0.03
1.44	2795695	1-Pentene, 2-methyl-	59	84.09	0.03
1.62	1500145	2-(Diethylamino)-N-(9,10-dioxoanthracene-2-yl)-ethanamide	9	336.15	0.01
15.11	3015455	Benzoic acid, 2-[(1,1-di(trifluoromethyl) propylamino](hydroxy) methylenamino-, methyl ester	25	372.09	0.03
15.96	1E+10	Ethanol, 2,2'-dithiobis-	96	154.01	94.79
18.09	10320233	Ethanol, 2,2'-dithiobis-	43	154.01	0.1
18.43	2379835	Ethanol, 2,2'-dithiobis-	91	154.01	0.02
18.52	4.91E+08	Ethanol, 2,2'-trithiobis-	76	185.98	4.65
20.45	5839524	Ethanol, 2,2'-trithiobis-	80	185.98	0.06
21.45	760616	Ethanol, 2,2'-trithiobis-	38	185.98	0.01
22.39	2663501	Heneicosane	76	296.34	0.03
23.29	4716506	Docosane	81	310.36	0.04
24.16	4446900	Tridecane, 1-iodo-	83	310.12	0.04
24.98	2790882	Tetracosane	95	338.39	0.03
25.06	990664	Hexanedioic acid, mono(2-ethylhexyl) ester	35	258.18	0.01
25.77	1585863	Hexadecane, 1-iodo-	72	352.16	0.02
26.26	1187146	2-(Octyloxycarbonyl)benzoic acid	59	278.15	0.01
26.53	697017	Undecane, 2,9-dimethyl-	53	184.22	0.01
30.44	2366189	3-Mercaptopropionic acid	38	106.01	0.02
31.09	3166017	(Z, Z, Z, Z)-6,9,12,15-Octadecatetraenoic acid, picolinyl ester	27	367.25	0.03

3.2 Conversion of SS

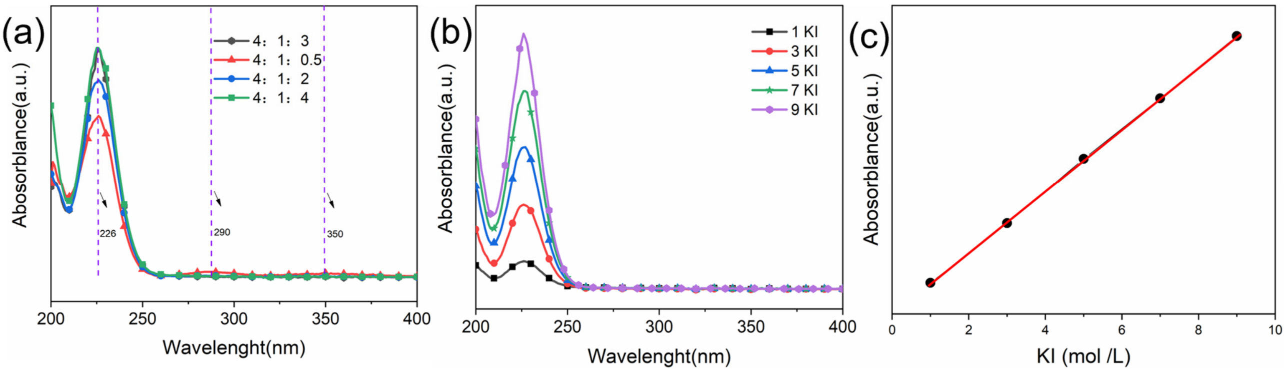

Figure S1. The UV-vis spectra of the synthetic solution at different stage of the reaction.

Prepare 10 ml of 4 mM potassium iodide aqueous solution and add 1 mmol iodine to obtain an aqueous solution of iodide tri - and iodide ions, the solution is reddish brown. Then 2 mmol of 2-Hydroxy-1-ethanethiol was added dropwise to the solution, (where the molar ratio of iodotriene to sulfhydryl was 1:2) as the oxidation of sulfhydryl groups to disulfide bonds by iodotriene ions changed the solution to colorless, and when added to 82% ~ 84%, the solution turned pale yellow, proving that the iodotriene was not reacting with sulfhydryl groups. Take the appropriate amount of test ultraviolet, from the ultraviolet, we can know the figure as potassium iodide different concentration plot, the lower figure is the concentration curve, and the standard curve equation is $y = 0.3001x \pm 0.0061$. Figure S1 (a) is the UV test graph of different concentrations of 2-Hydroxy-1-ethanethiol, Figure S1 (b) and (c) is the concentration curve of KI, it is known from the graph that when 2-Hydroxy-1-ethanethiol is small, the peak at 225 nm is weak because there is little iodide ion generated. The peaks at 288 nm and 350 nm are strong because they also contain iodide triion unreacted. When 2-Hydroxy-1-ethanethiol was in excess, the absorption peaks at 288 nm and 350 nm disappeared, proving the absence of iodinetriion. The appearance of weaker peaks at 288 nm and 350 nm with a 1:2 ratio of iodide Trio: sulfhydryl group proved to contain a small amount of iodide Trion, and taking the ki concentration curve from the intensity of the iodide peak at 222

nm resulted in the conversion of (1.649 = 0.3001x±0.0061) to (5.495 mmol/L) with (5.495-3)/3×100% = 83.16%.

3.3 Purity data from literatures

Table S2. Purity of disulfide preparation

Entry	solvent	oxidant	Catalyzer	Yield /%	Ref.
1	CH_2Cl_2	O_2	An_2Te	90	[17]
2	$AcOEt/H_2O$	O_2	$Cu(NO_3)\cdot 3H_2O$	98	[18]
3	C_2H_6O	H_2O_2	Ni-complex-boehmite I	95	[19]
4	CH_2Cl_2	Br_2	W/O	92	[20]
5	$H_2O:CH_3CN$	I_2	W/O	94[b]	[21]
6	H_2O	I_2	W/O	94.79[b]	This work

[b]Yield referred to isolated products..

3.4 H NMR

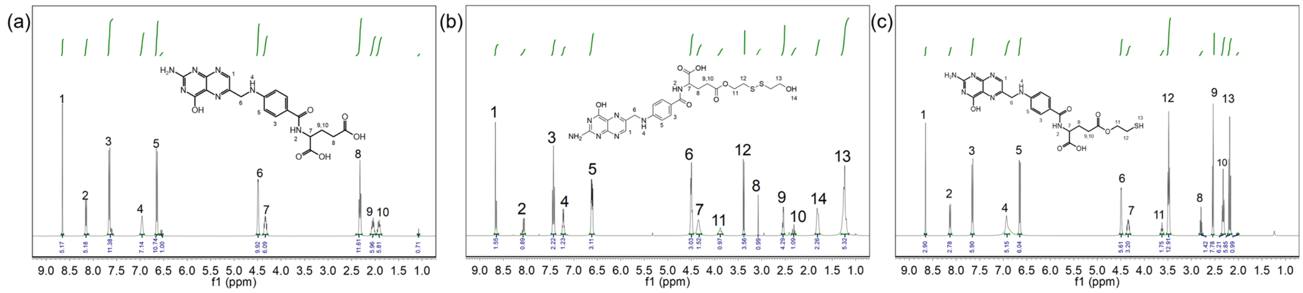

Figure S2. (**a**) 1H NMR Spectra of FA; (**b**) 1H NMR Spectra of Fa-SS; (**c**) 1H NMR Spectra of TFa.

3.5 Purity of TFa

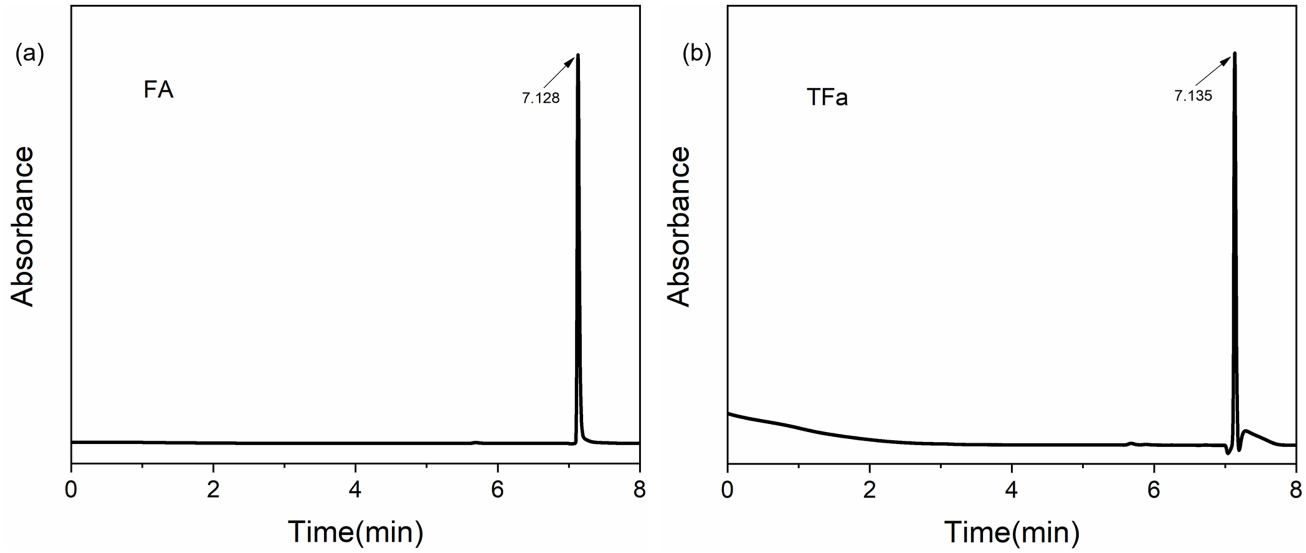

Figure S3. (**a**) HPLC of FA; (**b**) HPLC of TFa.

Table S3. The data from HPLC pattern of FA (Figure S3a)

No.	Retention Time/min	Area mAU*min	Height mAU	Relative Area%	Relative Height %	Amount
1	5.223	0.024	0.142	0.07	0.02	n.a.
2	5.690	0.360	2.214	1.07	0.25	n.a.
3	5.890	0.155	0.871	0.46	0.10	n.a.
4	6.242	0.271	0.891	0.81	0.10	n.a.
5	6.377	0.660	0.917	1.97	0.10	n.a.
6	7.128	32.034	889.266	95.61	99.44	n.a.
n.a.	n.a.	n.a.	n.a.	n.a.	n.a.	n.a.
Total:		33.503	894.301	100.00	100.00	

Table S4. The data from HPLC pattern of TFa (Figure S3b)

No.	Retention Time min	Area mAU*min	Height mAU	Relative Area %	Relative Height %	Amount
1	5.680	0.140	0.596	5.92	0.91	n.a.
2	5.885	0.082	0.581	3.48	0.89	n.a.
3	7.135	2.143	64.257	90.60	98.20	n.a.
n.a.	n.a.	n.a.	n.a.	n.a.	n.a.	n.a.
Total:		2.366	65.434	100.00	100.00	

3.6 Conversion of TFa

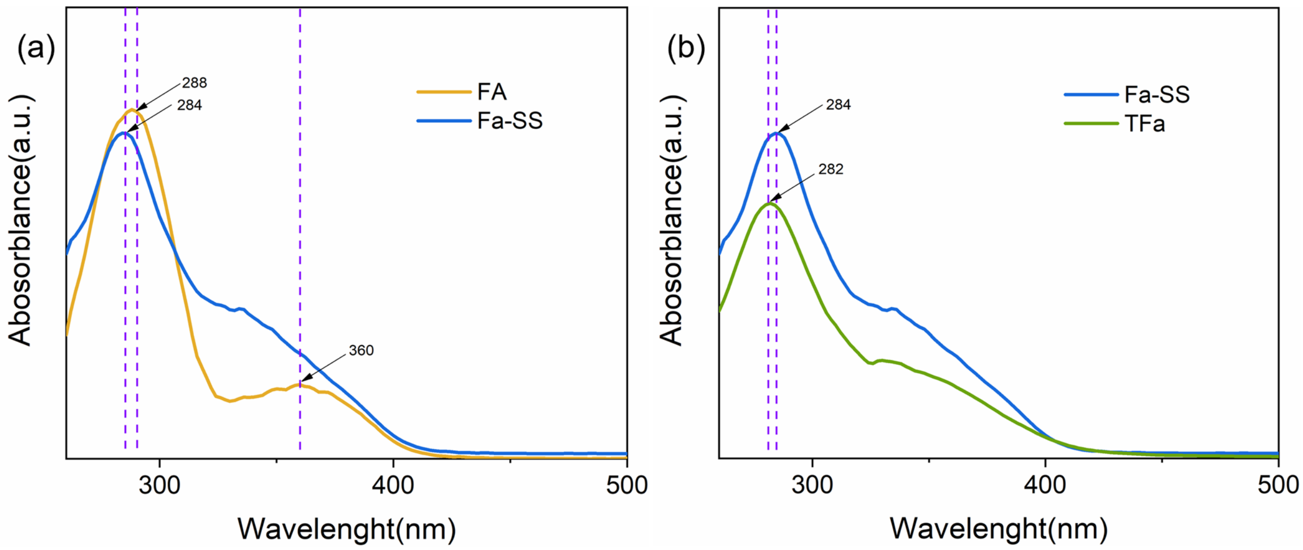

Figure S4. UV-Vis spectra of Fa-SS (**a**) and TFa (**b**).

FA-SS and TFa are dissolved in DMSO, respectively, and both concentrations are 10μM

Based on Figure S4 (a), the absorption spectrum of FA displays two distinct peaks at 288 nm and

360 nm. Upon reacting with SS, the peak at 288 nm undergoes a slight shift to 284 nm, accompanied by a minor reduction in peak intensity. Meanwhile, the peak at 360 nm experiences a significant reduction in absorption intensity. By Figure S4 (b), the addition of DTT results in the cleavage of disulfide bonds and the formation of TFa, the peak at 284 nm undergoes a slight shift to 282 nm. Based on the ultraviolet absorption intensity measurements, the calculated conversion rate of TFa is 73.19%.

3.7 FTIR spectrum of TFa modified AgNPs

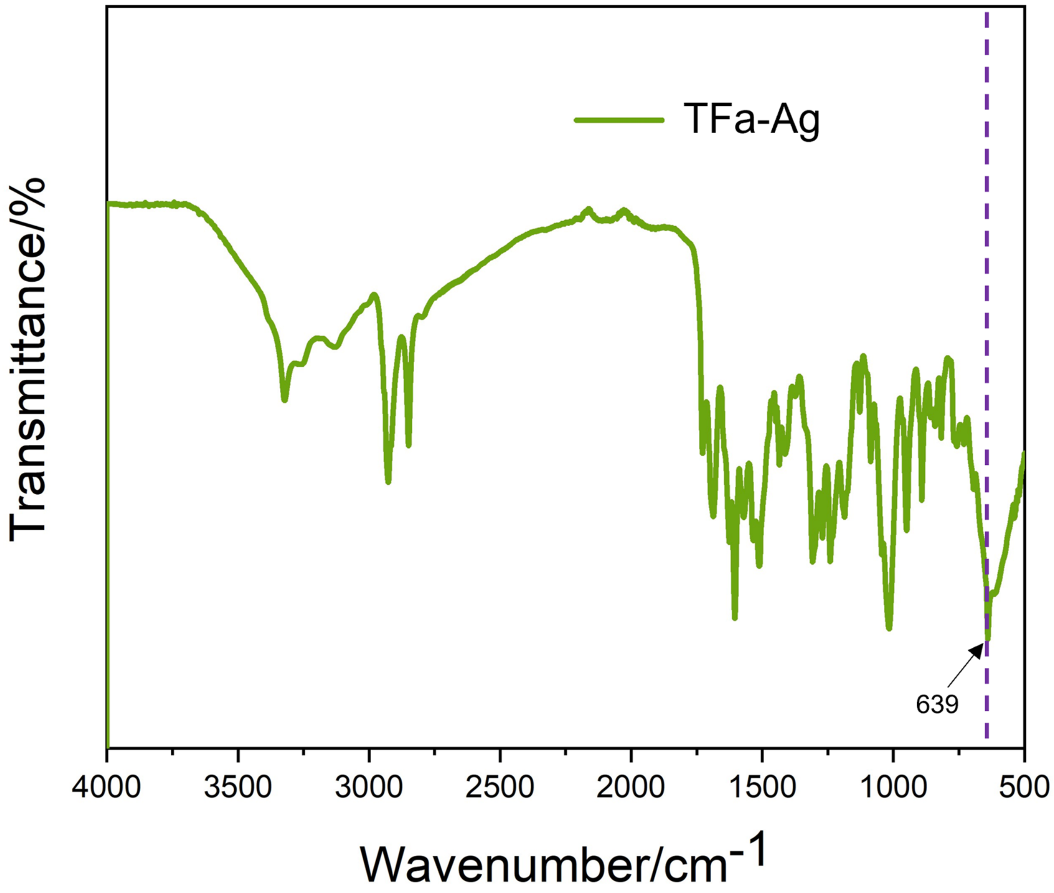

Figure S5. FTIR spectrum of TFa modified AgNPs

3.8 Melting point of Fa-SS and TFa

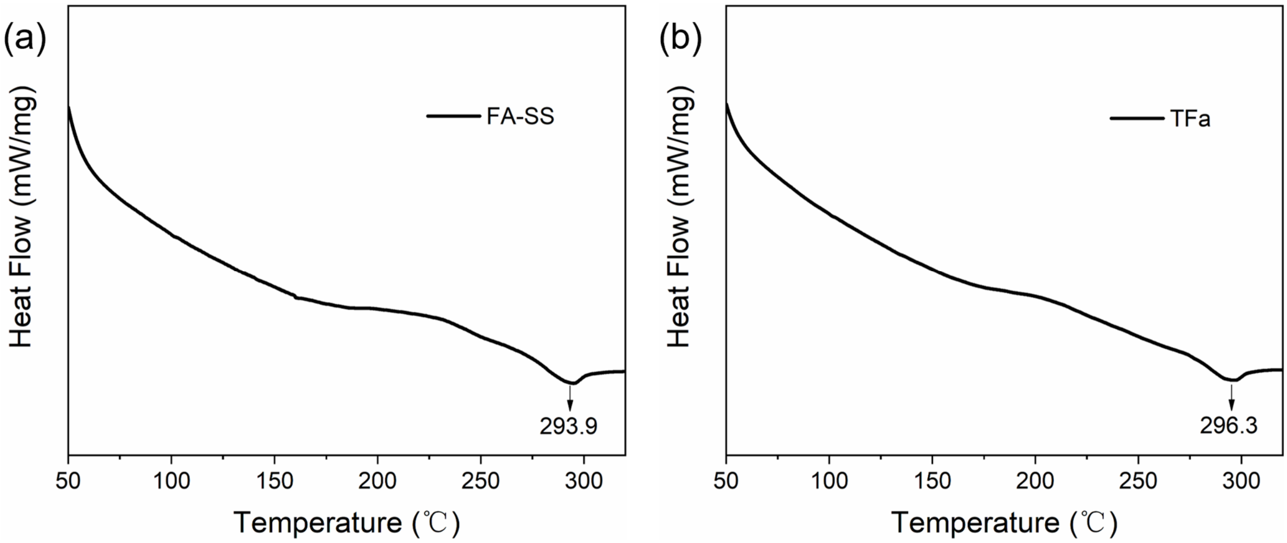

Figure S6. DSC patterns of Fa-SS (**a**) and TFa (**b**).

References

17. Soleymani, J.; Hasanzadeh, M.; Somi, M.H.; Shadjou, N.; Jouyban, A. Probing the specific binding of folic acid to folate receptor using amino-functionalized mesoporous silica nanoparticles for differentiation of MCF 7 tumoral cells from MCF 10A. Biosens. Bioelectron. 2018, 115, 61–69. https://doi.org/10.1016/j.bios.2018.05.025.
18. Nosrati, H.; Abbasi, R.; Charmi, J.; Rakhshbahar, A.; Aliakbarzadeh, F.; Danafar, H.; Davaran, S. Folic acid conjugated bovine serum albumin: An efficient smart and tumor targeted biomacromolecule for inhibition folate receptor positive cancer cells. Int. J. Biol. Macromol. 2018, 117, 1125–1132. https://doi.org/10.1016/j.ijbiomac.2018.06.026.
19. Wang, H.; Lin, S.; Wang, S.; Jiang, Z.; Ding, T.; Wei, X.; Lu, Y.; Yang, F.; Zhan, C. Folic Acid Enables Targeting Delivery of Lipodiscs by Circumventing IgM-Mediated Opsonization. Nano Lett. 2022, 22, 6516–6522. https://doi.org/10.1021/acs.nanolett.2c01509.
20. Huang, S.; Duan, S.; Wang, J.; Bao, S.; Qiu, X.; Li, C.; Liu, Y.; Yan, L.; Zhang, Z.; Hu, Y. Folic-Acid-Mediated Functionalized Gold Nanocages for Targeted Delivery of Anti-miR-181b in Combination of Gene Therapy and Photothermal Therapy against Hepatocellular Carcinoma. Adv. Funct. Mater. 2016, 26, 2532–2544. https://doi.org/10.1002/adfm.201504912.
21. Wang, X.; Xia, J.; Wang, C.; Liu, L.; Zhu, S.; Feng, W.; Li, L. Preparation of Novel Fluorescent Nanocomposites Based on Au Nanoclusters and Their Application in Targeted Detection of Cancer Cells. ACS Appl. Mater. Interfaces 2017, 9, 44856–44863. https://doi.org/10.1021/acsami.7b16457.
